# Protecting Persistent Dynamic Oceanographic Features: Transboundary Conservation Efforts Are Needed for the Critically Endangered Balearic Shearwater

**DOI:** 10.1371/journal.pone.0035728

**Published:** 2012-05-10

**Authors:** Maite Louzao, Karine Delord, David García, Amélie Boué, Henri Weimerskirch

**Affiliations:** 1 Centre d’Etudes Biologiques de Chizé, Centre National de la Recherche Scientifique UPR 1934, Villiers en Bois, France; 2 Department of Ecological Modelling, UFZ - Helmholtz Centre for Environmental Research, Leipzig, Germany; 3 Centro Oceanográfico de Xixón, Instituto Español de Oceanografía, Xixón, Spain; 4 SEO/BirdLife, Barcelona, Spain; 5 LPO/ Birdlife France - Service Etudes du Patrimoine Naturel, Rochefort, France; Hawaii Pacific University, United States of America

## Abstract

The protection of key areas for biodiversity at sea is not as widespread as on land and research investment is necessary to identify biodiversity hotspots in the open ocean. Spatially explicit conservation measures such as the creation of representative networks of marine protected areas (MPAs) is a critical step towards the conservation and management of marine ecosystems, as well as to improve public awareness. Conservation efforts in ecologically rich and threatened ecosystems are specially needed. This is particularly urgent for the Mediterranean marine biodiversity, which includes highly mobile marine vertebrates. Here, we studied the at sea distribution of one of the most endangered Mediterranean seabird, the critically endangered Balearic shearwater *Puffinus mauretanicus*. Present knowledge, from vessel-based surveys, suggests that this species has a coastal distribution over the productive Iberian shelf in relation to the distribution of their main prey, small pelagic fish. We used miniaturised satellite transmitters to determine the key marine areas of the southern population of Balearic shearwaters breeding on Eivissa and spot the spatial connections between breeding and key marine areas. Our tracking study indicates that Balearic shearwaters do not only forage along the Iberian continental shelf but also in more distant marine areas along the North African coast, in particular W of Algeria, but also NE coast of Morocco. Birds recurrently visit these shelf areas at the end of the breeding season. Species distribution modelling identified chlorophyll *a* as the most important environmental variable in defining those oceanographic features characterizing their key habitats in the western Mediterranean. We identified persistent oceanographic features across time series available in the study area and discuss our results within the current conservation scenario in relation to the ecology of the species.

## Introduction

The protection of key areas for biodiversity at sea is not as widespread as on land and research investment is necessary to identify biodiversity hotspots in the open ocean. It is now globally accepted that spatially explicit conservation measures such as the creation of representative networks of marine protected areas (MPAs) is a critical step towards the conservation and management of marine ecosystems, as well as to improve public awareness [1], [2]. Conservation efforts in ecologically rich and threatened ecosystems are especially needed, urgently for the Mediterranean Sea [2]. This marine ecosystem is particularly diverse showing both high degree of endemism (around 20–30%) [3] and high occurrence of threatened species [1], covering only 0.3% of the global oceans while hosting 7% of the world’s marine species [3]. Moreover, species richness shows a NW to SE decreasing gradient for both invertebrates and vertebrates related to important environmental drivers such as salinity, temperature, and water circulation, with a highly heterogeneous distribution depending on the region considered (highest for vertebrates around Sicily, northwestern coastal and shelf areas) [4]. However, current protected areas (namely MPAs) do not constitute a representative network since most of them are located in shallow waters of the northern part of the basin and represent 3.8% of the total surface of the Mediterranean Sea [1]. This may be a consequence of the limited marine research efforts of several eastern and southern regions of the Mediterranean [4], which could have delayed the implementation of protected areas in these biogeographic areas. Thus, ecologically important habitats of high conservation value (especially those identified in the southern and eastern Mediterranean), including pelagic habitats of highly mobile marine vertebrates [1], should be protected.

The Mediterranean Sea has been exploited and modified for thousands of years (e.g. fisheries) and it hosts very large populations of pelagic top predators - resident and transient (e.g. tuna, swordfish, dolphins, whales and seabirds) [2]. Upper-trophic level predators have been suggested as good indicators of the ecosystem functioning [5] while they integrate marine food webs across space and time [6]. Therefore, they provide information on ecosystem changes (i.e. variations in prey availability may influence breeding population sizes [7]). Seabirds are predators easy to monitor when visiting their land-based colony and the study of their at-sea distribution by using tracking devices is thus facilitated. They follow the distribution of their prey, which depends on abiotic and biotic factors interacting at different spatial and temporal scales [8]. Preys are patchily distributed, but different physical processes can retain prey at certain oceanographic features (e.g. eddies, river plumes, slope currents). Productivity at these oceanographic features can vary at different temporal and spatial scales and species can shift their distribution or habitat preferences due to environmental changes. However, year-to-year persistent productive areas are likely to be exploited recurrently by top predators over years [9], [10].

Here, we studied the at sea distribution of one of the most endangered Mediterranean seabirds, the Balearic shearwater *Puffinus mauretanicus*. Due to its small (ca. 3200 breeding pairs) and declining population (7.4% decrease per year [11]), this pelagic species is currently listed as Critically Endangered on the IUCN Red List [12]. It breeds solely on the Balearic Islands, on Menorca (northern populations), Mallorca-Cabrera (central populations) and Eivissa-Formentera (southern populations). Present knowledge from at sea vessel-based observations of birds of unknown origin, suggests that this species has a coastal distribution over productive shelf areas [13]. Oceanographic features promoting areas of high productivity influences the distribution of small pelagic fish, which constitutes the main natural prey for Balearic shearwaters apart from exploiting fishing discards [14]. During breeding (March–June), Balearic shearwaters forage along the Iberian continental shelf where different mesoscale oceanographic features result in productivity hotspots [13], [15]–[18]. They also forage around the breeding grounds in the Balearic archipelago (e.g. Menorca-Mallorca channel, South of Mallorca and the marine area surrounding Formentera and South of Eivissa) [13], [16], [18]. Furthermore, former satellite tracking data evidenced that Balearic shearwater from northern populations (Menorca) were visiting marine areas close to Algeria [16]. However, evidence was inferred from sparse locations of 3 tagged birds (out of 7) and another 3 tagged birds from central populations (Mallorca) foraged over the Iberian shelf and breeding sites, whereas there is no data available from southern populations (breeding in Eivissa-Formentera). Thus, more research is needed to confirm the importance of western North African waters for this critically endangered shearwater in the Mediterranean.

Our aim was to provide seminal information on the key marine areas of the southern population of Balearic shearwaters (the least known) to support conservation efforts by elucidating spatial connections between breeding and key marine areas. In a first step, we identify the main biogeographic areas where Balearic shearwaters from southern populations forage based on miniaturized satellite transmitters tracking data. Then, we develop species distribution models (SDM) in order to define those oceanographic features characterizing their key habitats in the western Mediterranean. We specially search for the persistence of identified oceanographic features across time series available in the study area. Finally, we discuss our results within the current conservation scenario and draw attention to the main threats for the species.

## Materials and Methods

### Tracking Data

Experiments comply with the current laws of the country, and were performed under the permission CAP03/2011 of the *Direcció General de Biodiversitat* (*Conselleria de Medi Ambient i Mobilitat, Govern de les Illes Balears*). The study was carried out on Illa de Conillera, Reserves Naturals des Vedrà, es Vedranell i els Illots de Ponent (west of Eivissa Island, western Mediterranean) during the 27^th^ and 28^th^ of May 2011. Tracking was conducted on adult Balearic shearwaters rearing large chicks in a cave where at least 10 nests were easily accessible. During this period, adults forage intensively in order to feed their chick while returning to the colony by night. In order to minimise our impact on chick provisioning and colony attendance, we decided to tag birds after they fed their chick while leaving the colony and capture them at the entrance of the cave just before they took off for the sea. Some non-breeding adults can also visit the colony at night at this period (as prospectors or early failed breeders) [19]. Therefore, the breeding status of the adults captured was only suspected as we did not directly observe chick feeding. We tagged 6 adults, 4 males and 2 females (sex was determined based on a species-specific discriminant function based on biometry measurements [20]). Adults weighed on average 525 g (range: 480 – 630 g). We deployed Argos satellite transmitters PTT (Platform Terminal Transmitters) solar panel, three 5 g and three 9 g-devices, with a duty-cycle of 48-hour off period, with 10-hour on period (Microwave Telemetry, Columbia). PTTs were attached on the back feathers using solely Tesa Tape®. This method allows a rapid deployment of the tag and avoids a medium to long term impact on the bird, since tags are lost with feathers when birds moult at the end of the breeding season. The total mass of devices was below the recommended 3% threshold (range: 0.9–2%) [21].

Previous to any analyses, we discarded those positions over land. Then, we used all Argos locations (accuracy classes A, B, 0, 1 to 3), after filtering positions above 70 km h^−1^ (McConnell, Chambers & Fedak 1992) which is the maximum GPS-based speed of the closely related Manx shearwater *Puffinus puffinus* [22]. Speed filtering led to the removal of the 27 % of the positions. We also explored an important feature of our tracking data, the duty cycle programme. Argos PTTs had a duty cycle programme (10 hrs on, 48 hrs off) that lead to alternating day-night transmission periods (see Text S1). After filtering the data, the number of locations per duty cycle ranged between 1 and 9 (Text S1). Indeed, transmission period started around 5 am and 2 pm, while no transmission started between 9 am and 10 am (Text S1). The number of 10-h on periods ranged between 8 and 12 with a range of 1.81 and 5.33 locations per duty cycle lasting between 0 and 10.09 hours (Table 1 & Text S1).

**Table 1:** Summary of birds (sex and weight), PTT devices (weight and date of equipment) and transmission characteristics (date of first and last position, number of locations, percentage of discarded positions, number of active duty cycles and mean (range) number of locations per duty cycle).

Shearwaters Status	Sex	WB (g)	PTT Number	WD (g)	Date ofequipment	Date of first position	Date of lastposition	# LOC	LOC over CONT (%)	DISC LOC (%)	# DC	# LOC/duty cycle mean (min - max)
Breeder	Male	610	40872	9	27/05/2011 21∶00	29/05/2011 20∶22	29/05/2011 21∶13	2	1 (50.0)	NA	NA	NA
Breeder	Male (?)	480	40866	5	27/05/2011 21∶30	30/05/2011 2∶57	08/07/2011 6∶10	30	2 (6.7)	2 (7.1)	11	2.36 (1–6)
Breeder	Male	630	40895	9	27/05/2011 22∶30	29/05/2011 19∶27	26/06/2011 10∶11	95	23 (24.2)	8 (11.1)	12	5.33 (3–8)
Breeder	Female	555	40825	5	28/05/2011 21∶00	29/05/2011 16∶53	29/06/2011 10∶16	27	3 (11.1)	4 (16.7)	11	1.81 (1–4)
Breeder	Male	490	40870	9	29/05/2011 0∶00	29/05/2011 19∶24	23/06/2011 13∶24	73	15 (20.5)	9 (15.5)	11	4.45 (1–9)
Non-breeder	Female	520	40823	5	29/05/2011 0∶00	01/06/2011 4∶13	19/06/2011 22∶17	36	12 (33.3)	4 (16.7)	8	2.5 (2–4)

WB: weight bird. WD: weight device. LOC: location. CONT: continent. DISC: discarded. DC: duty cycle.

Important marine areas were identified generating density distribution maps using fixed kernel density using the *ad hoc* method of the ‘adehabitat’ package (i.e. bivariate normal kernel; smoothing factor *h* of 0.51) and a cell size of 0.0417° (to match the spatial resolution of the satellite imagery data) in R 2.12.2 [23]. The smoothing factor was chosen based on exploratory analysis comparing the bivariate normal kernel, the least-square cross validation and arbitrarily chosen values (*h* = 1 and *h* = 2). The bivariate normal kernel method showed the best fit to our data of the western Mediterranean basin (see details in Text S2) and was further used for analyses. It is important to note that we explored the effect of the duty cycle on the kernel density estimates (see details in Text S3). The described duty cycle (10 hrs on, 48 hrs off) might provide locations in close proximity (during the 10 hr on-cycle) but then none for 2 days. Hence, the kernels may give more emphasis to the areas where the PTT transmitted, rather than properly reflect the proportion of time spent by the birds. We test this influence by using just one location per duty cycle (e.g. the centroid of all locations per duty cycle) and re-calculating the kernels to see whether they would change massively in size or location. Since kernel estimations did not change, we did not discard locations for further analysis. We also excluded the possibility of night-time activity periods heavily influencing the kernel density estimates (see Text S3).

### Environmental Predictors

We selected the most biologically relevant environmental variables from known habitat selection of the species [13], [24]. Oceanographic data were extracted corresponding to the central month of the study period (i.e. June 2011) for the western Mediterranean, from the Environmental Research Division, Southwest Fisheries Science Center and US National Marine Fisheries Service (http://coastwatch.pfel.noaa.gov/coastwatch/CWBrowserWW180.jsp). Dynamic oceanographic variables such as sea surface temperature (SST, °C, as a proxy of water mass distribution) and chlorophyll *a* concentration (CHL, mg m^−3^, as a surrogate of marine productivity) were extracted from MODIS. We extracted dynamic variables from each location from the corresponding raster of June 2011. Bathymetry (static variable; BAT, m, as a proxy of coastal versus pelagic domains) was extracted once. All variables were aggregated to match the standard grid (0.0417° cell size). Additionally, we estimated their spatial gradients by estimating their Proportional Change (PC) within a surrounding 3×3 cell (0.0417°×0.0417°) grid using a moving window as follows: PC  =  [(maximum value − minimum value) * 100] / (maximum value). This dimensionless metric expresses the magnitude of change in each habitat variable, scaled to the maximum value [13]. The spatial gradients of chlorophyll *a* (CHLG) and sea surface temperature (SSTG) indicate the presence of frontal systems, whereas the gradient of bathymetry (BATG) reflects the presence of topographic features (e.g. shelf break or seamount). To account for the influence of central-place foraging shearwaters [25], we included the distance between each grid cell and the colony (COLONY).

### Identifying Oceanographic Features Driving Shearwater Distribution

#### Data preparation

Prior to modelling, strongly ‘correlated’ (Spearman rank correlation coefficient, |r_s_|>0.6) predictors were identified by estimating all pair-wise Spearman rank correlation coefficients. This exploratory step is necessary since correlation between predictors might produce spurious results. High correlation was found for BAT-BATG and SST-COLONY pair-wise correlation coefficients. Therefore, we excluded BATG and SST from further analysis, based on previous knowledge on habitat selection of the species [13], [24].

#### Species distribution modelling

Species distribution model was performed with Maximum Entropy (MaxEnt) modelling based on only presence data (version 3.3.3 (http://www.cs.princeton.edu/~schapire/maxent/ [accessed 5 August 2011]) with default parameters (modelling script will be made available by email). It is considered one of the best modelling techniques [26] and uses background samples of the environment rather than absence locations to estimate environmental relationships. Background samples were drawn within the western Mediterranean (latitudinal range: 34.73°N–42.86°N; longitudinal range: 5.70°W–4.88°E) to facilitate a valid comparison with previous habitat models within the known foraging range of the species [13], during the central months of the study period (i.e. June 2011). Although Maxent can fit complex relationships to environmental variables, we chose to fit only linear and quadratic relationships due to the difficult interpretation of other more complex relationships. Maxent is robust to small sample sizes [27], with spatial positioning errors [28], and to spatial mismatch between locations and environmental variables [29], [30]. Moreover, it gives the probability distribution of maximum entropy taking into account data available on species distribution and the environmental conditions across the study area [31], [32].

To minimise the influence of any individual on the population-wide model, we randomly selected an equal number of locations for each bird based on a boostrapping procedure [30]. It is important to note that we developed habitat models for breeding birds and, in turn, we did not include locations of the suspected non-breeder. The number of locations per bird was determined by the minimum value after applying the speed filter (20 locations), totalling 80 locations for developing species distribution models. Therefore, we took 100 random draws for those birds that had more than 20 locations without replacement. Due to the duty cycle of the tracking devices, autocorrelation of satellite positions was limited and no more filtering was necessary [30]. We ran Maxent on the randomly selected satellite positions 100 times. We calculated the mean of the 100 Maxent predictions to obtain an average prediction and coefficient of variation of predictions [30]. In addition, we evaluated the contribution of the environmental variables to the Maxent model based on a jackknife procedure, providing the explanatory power of each variable when used in isolation.

#### Model evaluation

To assess the predictive performance of SDMs, we evaluated each Maxent prediction using the Area Under the receiver operating characteristic Curve (AUC) [33]. AUC evaluates how well model predictions discriminate between locations where observations are present and absent (i.e. the presence locations and background in our study). AUC can range from 0 to 1. An AUC of 0.5 indicates that model performance is equal to that of a random prediction, whereas values from 0.5 to 1 with the following model predictive performance classification: >0.9 excellent, 0.9–0.8 good, 0.8–0.7 reasonable, 0.7–0.6 poor and 0.6–0.5 unsuccessful [34]. We applied two cross-validation procedures running 100 replicates: (1) an internal validation using data only from Eivissa (i.e. using original data) and (2) an optimal validation using an independent dataset [35].

In the case of the internal validation, we randomly selected 80 locations and trained the model, while the resulting model was tested on the remaining 79 locations (total sample size of 159 locations). Regarding the optimal solution, an independent tracking dataset was available for birds from northern and central populations (Menorca and Mallorca, respectively) tagged 10 years ago (June 1999–2000) [4]. We developed a SDM with data from Eivissa in predicting distribution patterns of birds from Menorca (the population with enough sample size). Working on two spatially distinct groups (Eivissa and Menorca) allowed us to assess the model transferability in time and space. SDMs were trained with data from Eivissa and the model was then used to predict the distribution of birds from Menorca.

For both evaluation procedures, the AUC was estimated for each simulation and the mean, upper and lower 95% confidence interval (CI) of the AUC were used as a cross-validation measure of the predictive performance of the models [36]. If the lower 95% CI limit did not include the 0.5 value, there was evidence that SDMs were able to accurately predict beyond training dataset.

### Oceanographic Persistence at Key Marine Areas

Once key marine areas were identified, we intended to estimate the persistence of significant dynamic oceanographic features. After distribution modelling (see Results Section), chlorophyll *a* was found to be the most important dynamic environmental variable. Since the presence probability of Balearic shearwaters increased with this dynamic variable, we quantified its persistence across the study area during the annual mean to assess its predictability for key marine areas. We hypothesised that those marine areas consistently identified as productive (indicated by high concentration of chlorophyll *a*) might be visited by Balearic shearwaters (and marine top predators in general) from one year to the next.

We extracted the longest time series of chlorophyll *a* (July 2002–July 2011). General persistence across the study area was analysed using a 3-step approach. Firstly, we obtained an average annual map and identified those pixels with high nutrient concentration (CHL >0.3 mg m^−3^) [37]. Then, we counted for each pixel in how many years (maximum of 8 corresponding to the CHL time series) the concentration corresponded to high or low concentration. In addition, we extracted monthly CHL values from the main key areas identified by kernel analysis to compare oceanographic conditions between geographically distant important marine areas.

## Results

### Satellite Tracking and Kernel Analysis

A total of 263 satellite positions were recorded from the 6-tagged shearwaters during the transmitter emission period (range: 0.04–39.13 days). Summary statistics of tracking devices can be found in Table 1. One of the PTTs provided only 2 locations over the Iberian continental shelf. From the remaining 5 PTTs, we discarded between the 6.7 % and 33.3 % of locations that were over land from which we disregarded between the 7.1 % and 16.7 %, after applying the speed filter. Overall, we discarded the 13.1% of the total locations after filtering by the speed threshold. Tracking devices were lost when birds started moulting, which took place between the 19^th^ June 2011 and 8^th^ July 2011.

**Figure 1:**
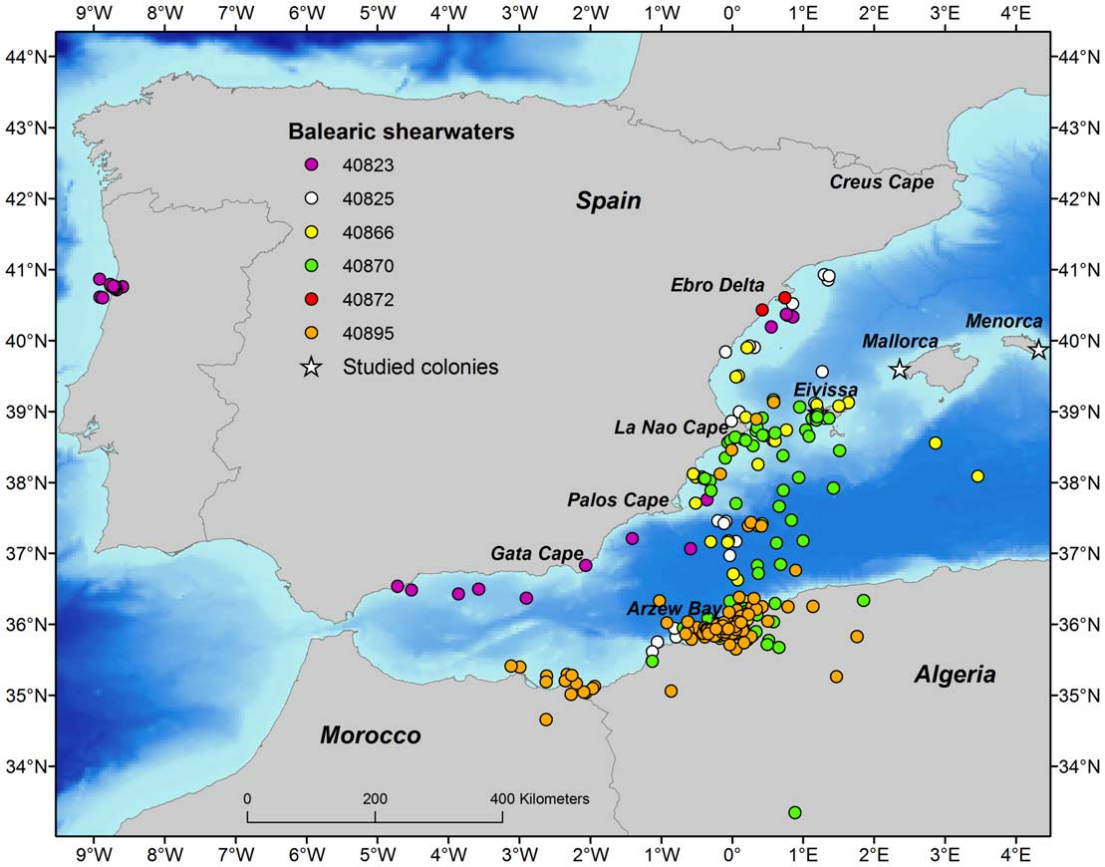
Satellite tracking of Balearic shearwaters during the chick-rearing period of 2011 from a southern breeding population (Conillera Island, western Mediterranean). Breeders stay in the western Mediterranean basin, whereas one non-breeder travels and stays in Portugal (violet points). Background values represent depth. Studied colonies are represented by white triangles.

Breeders remained in the western Mediterranean basin, only one -suspected non-breeder-individual travelled to Portuguese coastal waters (PTT 40823, see Figure 1). After deployment on the 29^th^ May 2011 night, this shearwater foraged over the southern sector of the Ebro Delta 3 days later (1^st^ June 2011) and 2 days later (3^rd^ June 2011) was entering the Atlantic Ocean through the northern coast of the Strait of Gibraltar, travelling more than 400 km from Cape Palos to Málaga in only 8 hours. Then, the same bird reached the northern coast of Portugal (Aveiro) on the 5^th^ June 2011 after at least 2 days travelling 900 km and stayed until the last position on the 19^th^ June 2011 (see Figure 1).

**Figure 2:**
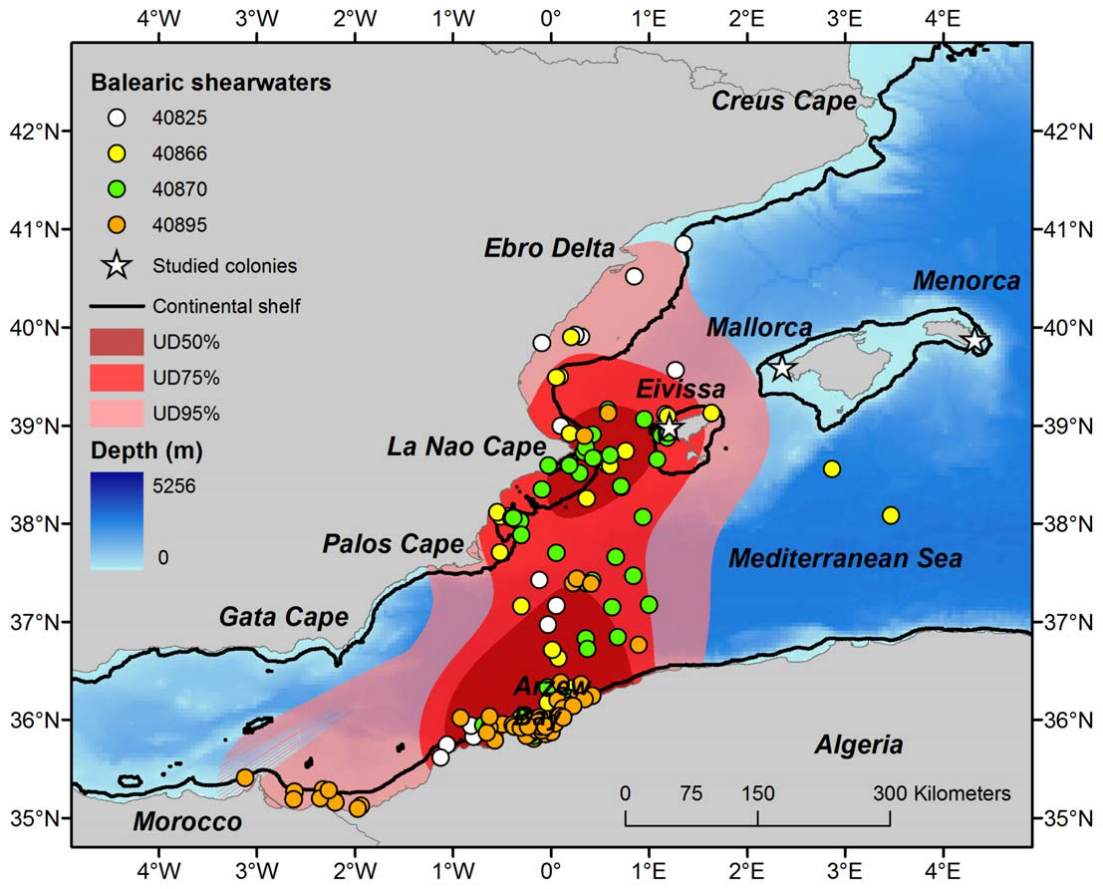
Filtered locations (each shearwater represented by different colours) and density contours (95%, 75% and 50% UD represented by black, dark grey and light grey lines, respectively) resulting from kernel estimation of the distribution of southern Balearic shearwaters during the chick-rearing period of 2011. Two main marine areas (indicated by the 50% UD) are identified: marine area around Cape La Nao and Arzew Bay in the Iberian and Algerian continental shelves, respectively.

Breeders commuted between the colony and several areas of shallow waters, the Iberian Peninsula and also the Algerian and Moroccoan waters (Figure 1). Filtered positions were assigned to different biogeographic areas (Figure S1): continental shelf around the colony (Eivissa, EIV), Iberian Peninsula (IBE), Algeria (ALG) and Morocco (MOR), as well as commuting between continental shelves. Commuting between Eivissa or Iberian and Algeria lasted on average 6 h and 20 min, mainly during daylight (n = 4; see examples in Figure S2). The Algerian continental shelf accounted for the highest number of locations, followed by the Iberian Peninsula and commuting (represented by the percentage of locations in each biogeographic area) locations. One bird (40895) strongly influenced this pattern since locations of this bird represented 75% of locations in Algeria. This individual also visited the eastern coast of Morocco. Kernel analysis identified two main key marine areas (indicated by the 50% UD) for southern Balearic shearwaters: marine area around Cape La Nao and Arzew Bay in the Iberian and Algerian continental shelves, respectively (Figure 2).

### Shearwater Distribution Modelling

**Figure 3:**
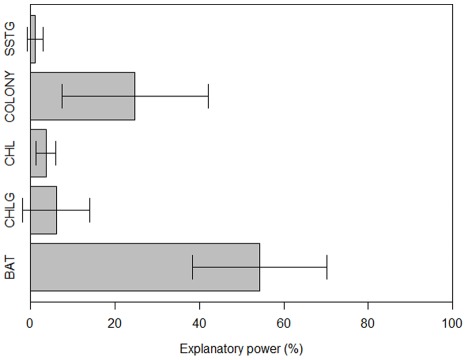
Variable importance estimated by the jackknife test. Bars indicate the explanatory power (in terms of gain) when only a single predictor is included in the model with 95% confidence intervals.

**Figure 4:**
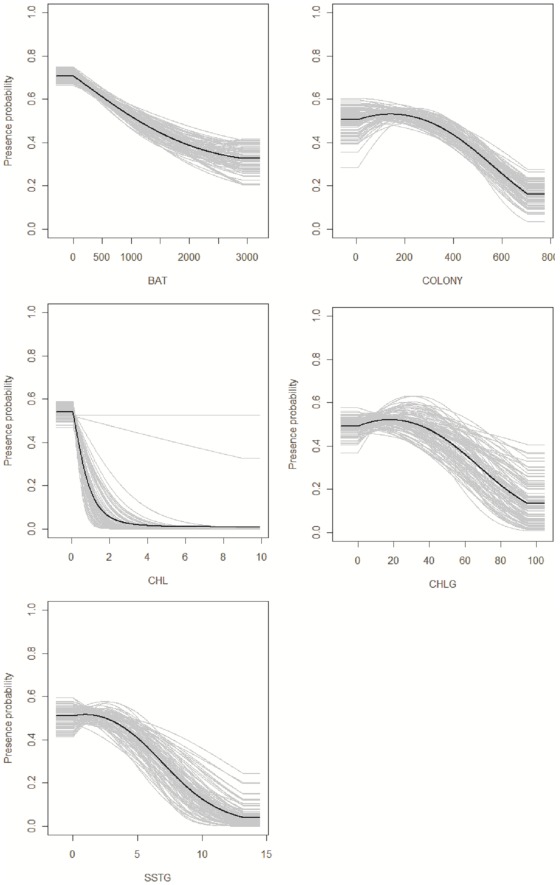
Response curve illustrating relationship of presence probability to environmental variables. Higher values correspond to higher probability of occurrence. These curves show how the logistic prediction of a particular variable changes, keeping all other environmental variables at their average sample value. Grey lines show the output of the 100 iterations, while the black line represents the mean bootstrap value.

Distribution models for southern Balearic shearwaters yielded reasonable model performance (AUC (CI 95%): 0.70 (0.66–0.73)). The variables that most contributed to explain southern shearwater distribution were BAT followed by COLONY, CHLG and CHL (Figure 3). Regarding response curves, the relationship between presence probability and environmental variables differed as illustrated in Figure 4. Shearwater presence probability decreased linearly with BAT, whereas non-linear relationships were found for remaining variables. Maximum presence probability was found at 200 km from the colony, lower CHL values, 20% of CHLG and 1% of SSTG. In summary, presence probability was higher over the continental shelf to a certain distance to the colony in association to frontal systems of productive areas.

Model predictions matched observed patterns within the range of southern Balearic shearwaters and identified key marine areas beyond the training dataset in June 2011.

**Figure 5:**
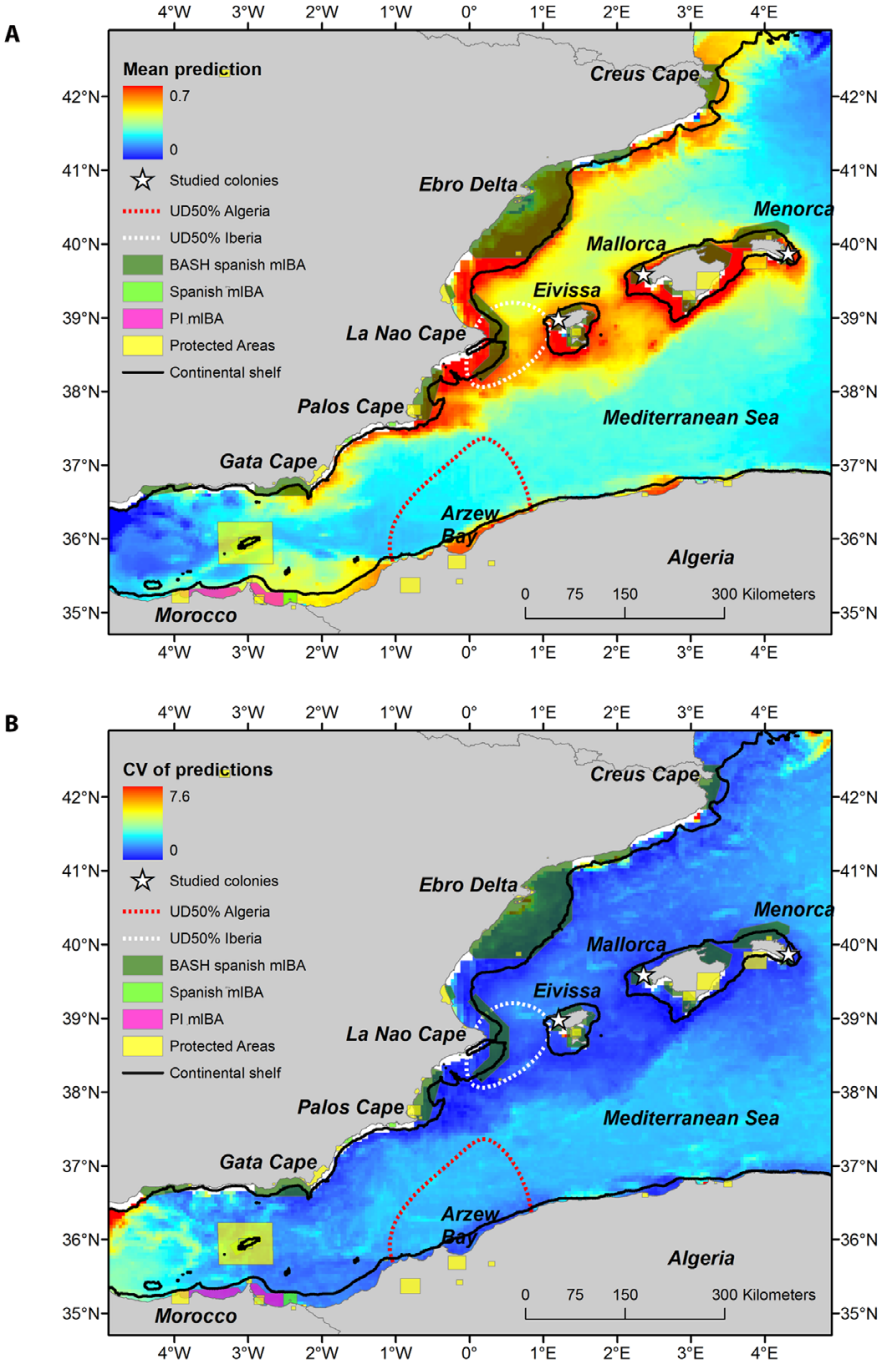
Species distribution modelling output for southern Balearic shearwaters distribution in June 2011: (a) mean prediction and (b) coefficient of variation. The position of the studied colony (white triangle), the two key marine areas (indicated by the 50% UD) around Cape La Nao and Arzew Bay, and the 200 m isobath (i.e. the limit of the continental shelf) are shown (black line). In addition, limits of current Protected Areas are placed by yellow polygons in the western Mediterranean [56], as well as Spanish marine Important Bird Areas (mIBAs) for Balearic shearwaters (BASH) (dark green polygons), other Spanish mIBAs (light green polygons) and potentially important (PI) mIBAs in international waters (violet polygons) [18].

Shearwaters were found with higher probability along three continental shelves: Balearic Islands (i.e. breeding colonies), Iberian and Algerian coast (dark red areas in Figure 5a). Those areas were consistently identified as important due to the low coefficient of variation in predictions (Figure 5b). Around breeding sites, all marine areas showed high presence probability excepting the North coast of Mallorca and Menorca. Along the Iberian continental shelf, presence probability was higher from the Ebro Delta until Cape Palos, as well as from Nador (Morocco) to Argel (Argelia) along the narrow continental shelf of North Africa.

Regarding model evaluation, the internal validation showed a reasonable model performance (AUC (CI 95%): 0.70 (0.67–0.74)), as well as the external validation (AUC (CI 95%): 0.67 (0.64–0.71). Thus, the SDM developed with data from Eivissa had the ability to predict distribution patterns from birds from Menorca.

### Productivity Persistence at Key Marine Areas

**Figure 6:**
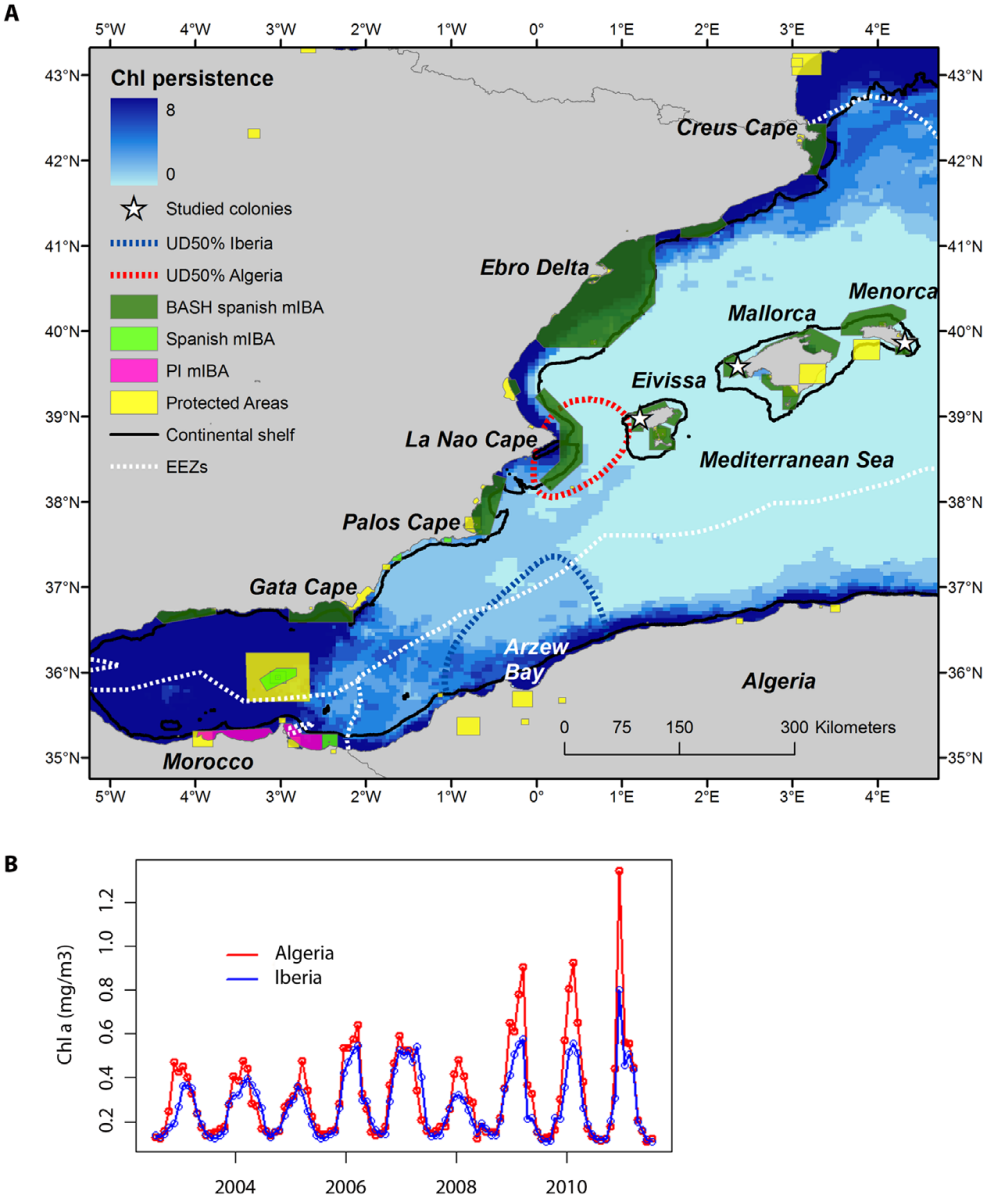
Oceanographic persistence at key marine areas. (a) Persistence of chlorophyll *a* values in the western Mediterranean during the breeding period (February–June) of Balearic shearwaters. Values were obtained from the MODIS sensor between July 2002 and July 2011. For each pixel, we counted in how many years (maximum of 8) the concentration corresponded to high nutrient concentration (>0.3 mg m^−3^). Bluer areas correspond to persistent productive areas (check number of years on the scale bar). The position of the studied colony (white triangle), and the two key marine areas (indicated by the 50% UD) around Cape La Nao and Arzew Bay are placed. Limits of the continental shelf (200 m isobath) are defined by the black line, whereas Economic Exclusive Zones are delimited by dotted white lines [57]. In addition, limits of current Protected Areas are placed by yellow polygons in the western Mediterranean [56], as well as Spanish marine Important Bird Areas (mIBAs) for Balearic shearwaters (BASH) (dark green polygons), other Spanish mIBAs (light green polygons) and potentially important (PI) mIBAs in international waters (violet polygons) [18]. (b) Time series of chlorophyll *a* anomalies estimated for the two main marine areas based on kernel analysis. For each key marine area, we computed the chlorophyll *a* anomaly by estimating the global mean value and subtracting the global mean value to corresponding month.

We quantified the persistence of the most important dynamic variable (CHL) across the study area over 8 years (Figure 6a). We found that high CHL values (>0.3 mg m^−3^) occurred consistently along the coastal areas of the study area (bluer areas), with a break South of the Cape La Nao until the Alboran Sea. The central-southern parts of the western basin were the least persistent areas (clearer areas). Within the distribution range of Balearic shearwaters, areas with high presence probability matched highly predictable productive areas (compare Figure 5a and 6a). Regarding the two core areas identified by kernel analysis (areas covered by red lines in Figure 6a), CHL values in Algeria were on average 14% higher than in Iberia, especially after 2009 (Figure 6b). Seasonal patterns were similarly reproduced in both biogeographic areas.

## Discussion

### The Algerian Current: An Important Dynamic Feature for Balearic Shearwaters

Our tracking study clearly indicates that Balearic shearwaters do not only forage along the Iberian continental shelf, but also in more distant key marine areas located along the northern African coast, as suggested by early satellite tracking from Menorca [12], [16]. In fact, birds from Eivissa (located closer to the North African coast than Menorca) recurrently commute to the northern African coast (especially the Algerian coast, but also Morocco) at the end of the breeding season. The reason why shearwaters from Eivissa do not only forage over the closest productive area along the Iberian continental shelf could be related to the marine productivity of the western northern African coast and the relative proximity from the colonies (300 km). The dynamic of the Atlantic inflow along this biogeographic area is characterised by the Algerian Current where mesoscale instabilities create zones of enhancement of productivity [38]. In this area, chlorophyll *a* concentration is higher at cyclonic eddies linked to higher zooplankton abundance [39]. Across the current, large horizontal gradients exist in hydrography (e.g. salinity) as well as in phytoplankton and zooplankton biomass and species composition [40], [41]. Productivity is particularly high at the offshore band of the Algerian Current where high concentrations of nutrients and chlorophyll *a* are related to high zooplankton biomass levels and abundances [38], [42].

Few data are available on the distribution of the main prey of Balearic shearwaters along the Algerian coast (e.g. small pelagic fish [14]). Anchovy *Engraulis encrasicholus* and sardine *Sardina pilchardus* represent the most important small pelagic fish target for Algerian fleet (in terms of biomass) [43], but captures exhibited great fluctuations during the 1970–2008 period (Figure S3). Since landing data on small pelagic fishes can be used as a proxy of their availability in the system [44], we compared total landings of anchovy, sardine and sardinella *Sardinella aurita* for Algeria and Spain to compare prey availability between Iberian and Algerian continental shelves [45]. Overall, all three species seemed to be available in the system over the 1970–2008 period and slight differences might be due to differences in number of vessels and technological development.

The present study complements the broader ecological perspective of foraging movements of Balearic shearwaters providing information on a less studied area. From previous studies, we know that breeding Balearic shearwaters repeatedly exploit the same foraging grounds along the Iberian continental shelf in relation to predictable resources (i.e. mesoscale oceanographic features) [13], [15], [17], [24], [46]. Indeed, central-place foraging Balearic shearwaters continually commute between the less productive waters around the breeding colonies (Balearic Islands) and the highly productive waters of the shelf-slope areas of the Iberian Peninsula and the northern African coast (Figure S2). Commuting seems to be a common type of movement of pelagic birds within temperate and polar regions, and might suggest that breeding seabirds ‘know’ where to find food, probably from previous experience [47]. Within a coarser temporal and spatial scale, prey patches are likely to be scattered within mesoscale features [47] shifting depending on physical and biological drivers (e.g. river run-off, small pelagic fish spawning, fishing) [48].

### Transboundary Conservation Target Areas

Balearic shearwaters appear to favour shelf areas with dynamic oceanographic features. While static systems can easily be protected by designating areas of interest, dynamic habitats (e.g. eddies, frontal systems, currents) are much more problematic for conservation management. By combining tracking studies and habitat modelling, now it is possible to define the location of key marine areas, provided that models show a reasonable predictive performance and the validation with additional observations support model predictions. Within the process of identifying ecologically important marine areas, once key areas are located the next step is their protection. For southern Balearic shearwaters, key marine areas appear to lie mainly under the jurisdiction of Spain and Algeria (i.e. Exclusive Economic Zone, see Figure 6a) [12].

Given Balearic shearwater threatened status, extending protective measures beyond the breeding sites to the marine environment should be a priority, and marine zoning is essential in this way to prioritise those areas that are preferentially used by the species. Key marine areas over the Iberian continental shelf (e.g. centred in Cape Creus, Ebro Delta and Cape La Nao) have been already identified as marine Important Bird Areas (IBAs) for the Balearic shearwater and other seabird species (Figure 6a) [18], and are currently in the process of designation as marine Special Protection Areas (SPAs) by the Spanish government, in commitment with the European Union Bird Directive (2009/147/EC) (http://www.magrama.es/es/biodiversidad/participacion-publica/PP_borrador_orden_zepa_marinas.aspx). Once finally designated, these marine SPAs will automatically be considered as part of Natura 2000, the network of protected sites across the European Union. These areas would not only protect targeted study species, but also their underlying habitat. It contrasts highly with the southern Mediterranean waters. In the case of Balearic shearwaters, there was little information on the likely location of key marine areas along the North African coast. Thus, this is the first study identifying key marine areas for the conservation of Balearic shearwaters in the south-western Mediterranean Sea from Nador (Morocco) to Alger (Algeria). Southern Mediterranean key areas should be incorporated into a transboundary conservation initiative to constitute an effective network of protected sites to be considered in further Marine Protected Areas proposals such as Specially Protected Areas of Mediterranean Importance.

In these areas, it would first be important to obtain information on the interactions between shearwaters and the local fisheries, to identify potential threats such as by-catch. In a second step, conservation measures should include a compulsory fishery observer program to record potential bycatch level since direct observations are important to detect threat by fishing. Quantification of Balearic shearwater bycatch is sparse and (limited) observer programmes often have reported low bycatch rates, but when bycatch occurs up to 50 birds or more can get entangled in a single line, due to the gregarious behaviour of the species [49], [50]. This is a serious and direct threat for Balearic shearwaters given the high overall mortality experienced by the species [11].

Such dramatic incidental captures occurred in the Spanish Mediterranean bottom longline vessels [51]–[53] and Portuguese purse-seiners [12], and most likely also occur in other areas. The puzzle still gets more complicated when considering interaction between fisheries: seabird bycatch in both pelagic and demersal Mediterranean longline vessels increases when trawlers are not operating (i.e. less discards are available) [47], [52], pointing towards the need of an integrated multi-fisheries management approach [12].

Even if some information on bycatch is available, still more research is needed for the northern Mediterranean since systematic surveys are necessary in order to clearly identify the spatio-temporal window of those interactions. Perspectives are still worse for the southern Mediterranean, where there is no information available for bycatch [53]. In fact, due to the current severe conservation concern of the species the Spanish Government has now advised the inclusion of Balearic shearwater in the Agreement on the Conservation of Petrels and Albatrosses (ACAP) [54], which is a multilateral agreement for the conservation of Southern Hemisphere species. In fact, it will be the first European species included in this agreement. Thus, we highlight the urgent need to assess the interaction of Balearic shearwaters with the longline fishery within their key marine areas not only during breeding in the western Mediterranean, but also during the post-breeding period along the Mediterranean and North Atlantic [55].

### Conclusion

Transboundary conservation efforts are needed for the critically endangered Balearic shearwater. This is the main conclusion of the present study which tracked for the first time breeding birds from the southern population. Highly mobile animals are not attached to administrative boundaries and it is therefore essential to overcome legal limitations to provide full protection to highly threatened species. Within this framework, our results match previous studies in identifying similar key marine areas for the species over the Iberian continental shelf and improve our understanding by identifying key marine areas for Balearic shearwaters in southern Mediterranean waters. Along this biogeographic area, kernel analysis identified Arzew Bay as key marine area for tagged birds. Thanks to SDMs, we additionally were able to predict Balearic shearwaters distribution beyond observed data identifying all close bays along the northern African coast from Nador (Morocco) to Alger (Algeria) as potential key marine areas. These southern Mediterranean key areas could be integrated into a supranational conservation initiative to develop a successful network of protected sites across Mediterranean waters. We therefore highlight the importance of tracking studies and the establishment of long-term studies in order to comprehend how the current changing environment will impact on the distribution of species of high conservation concern in the future.

## Supporting Information

Figure S1 **Biogeographic area visited by each breeding shearwater.**
(DOC)

Figure S2 **Examples of a Balearic shearwater commuting.**
(DOC)

Figure S3 **Time series of small pelagic fish captures in the western Mediterranean.**
(DOC)

Text S1 **Duty cycle transmission.**
(DOC)

Text S2 **Choosing a smoothing factor for kernel analysis.**
(DOC)

Text S3 **Effect of duty cycle on kernel estimation.**
(DOC)
