# Supplementary material for: Protecting Persistent Dynamic Oceanographic Features: Transboundary Conservation Efforts Are Needed for the Critically Endangered Balearic Shearwater
Source: PLoS One. 2012 May 10;7(5):e35728. doi: 10.1371/journal.pone.0035728 (PMC3349676; doi:10.1371/journal.pone.0035728)
Supplement: Figure S1 — Biogeographic area visited by each breeding shearwater. (DOC) [file pone.0035728.s001.doc]

Fig. S1 Biogeographic area visited by each breeding shearwater (and the total) represented as the percentage of filtered locations that shearwaters spent over the continental shelves of Eivissa (EIV), Iberian Peninsula (IBE), Algeria (ALG) and Morocco (MOR), as well as commuting between continental shelves (COM).
