# Supplementary material for: Protecting Persistent Dynamic Oceanographic Features: Transboundary Conservation Efforts Are Needed for the Critically Endangered Balearic Shearwater
Source: PLoS One. 2012 May 10;7(5):e35728. doi: 10.1371/journal.pone.0035728 (PMC3349676; doi:10.1371/journal.pone.0035728)

Fig. S2 Examples of a Balearic shearwater commuting from Eivissa/Iberian Peninsula (around Cape La Nao) to Algeria. Red points represent discarded locations after speed filtering, whereas blue and black points represent day and night locations.


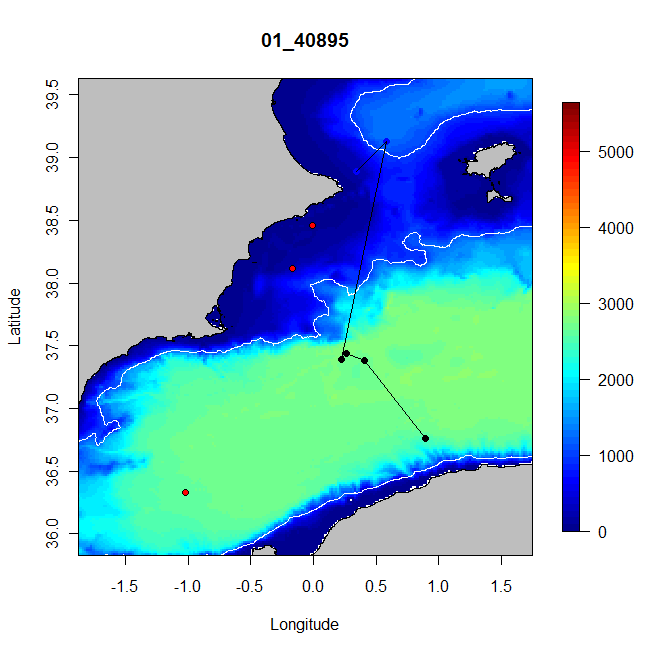

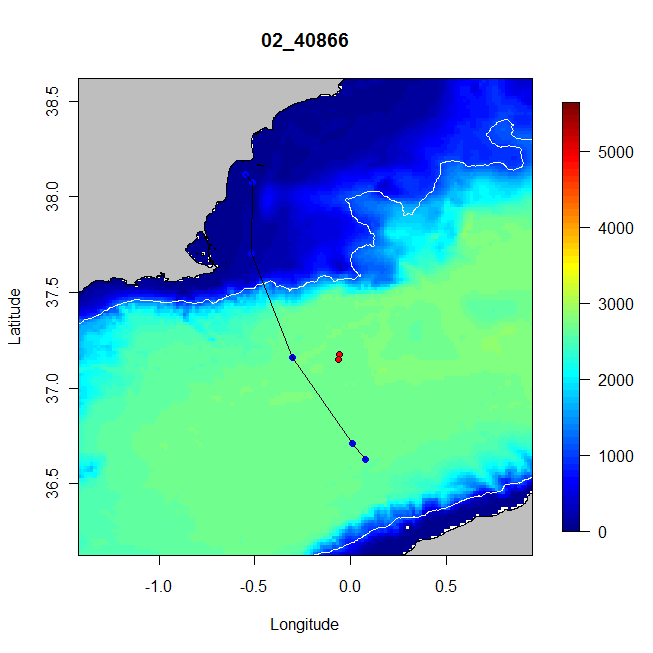


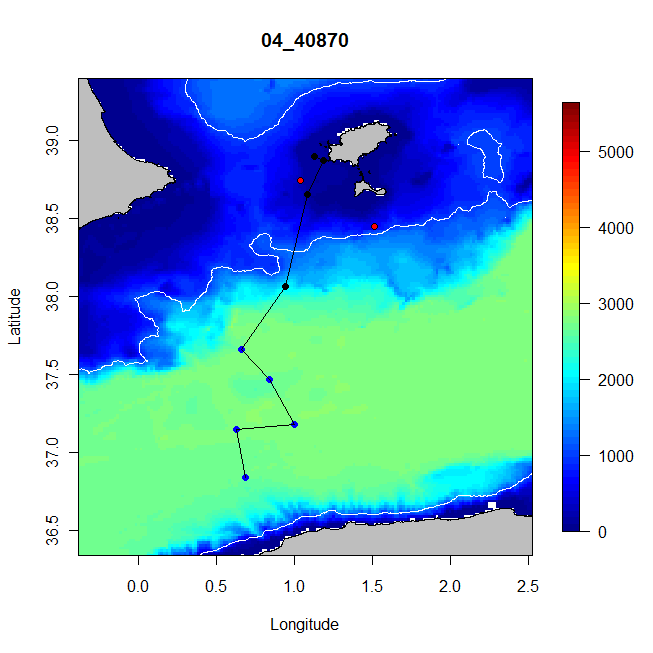

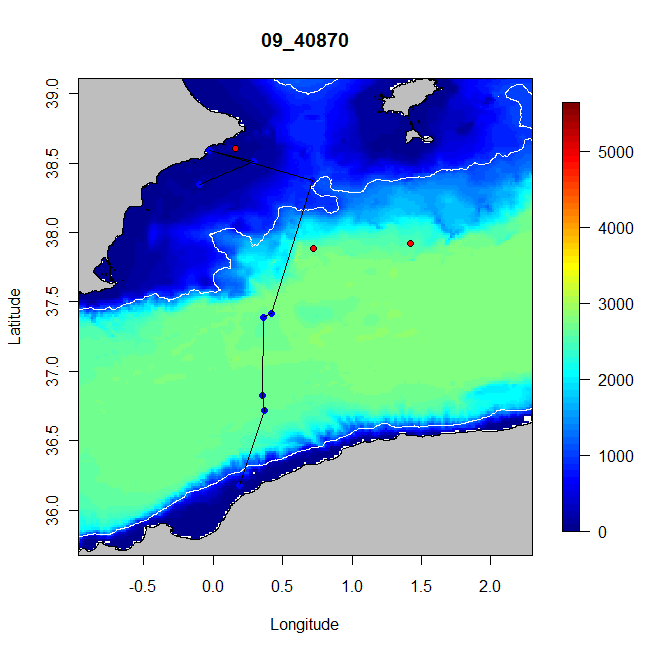

Supplement: Figure S2 — Examples of a Balearic shearwater commuting. (DOC) [file pone.0035728.s002.doc]
