# Supplementary material for: Protecting Persistent Dynamic Oceanographic Features: Transboundary Conservation Efforts Are Needed for the Critically Endangered Balearic Shearwater
Source: PLoS One. 2012 May 10;7(5):e35728. doi: 10.1371/journal.pone.0035728 (PMC3349676; doi:10.1371/journal.pone.0035728)
Supplement: Figure S3 — Time series of small pelagic fish captures in the western Mediterranean. (DOC) [file pone.0035728.s003.doc]

Fig. S3 Time series of small pelagic fish (anchovy, sardine and sardinella represented by circles, triangles and rectangles, respectively) captures in the western Mediterranean by Spanish and Algerian fishing fleets [1]. Captures for each country (red-Algeria and blue-Spain) are used as a proxy for prey availability over the two different foraging grounds located in Spain and Algeria of the southern Balearic shearwaters.

1. FAO (2011) FishStatJ: Universal software for fishery statistical time series. Available at http://www.fao.org/fishery/statistics/software/fishstatj/en.
