# Supplementary material for: Protecting Persistent Dynamic Oceanographic Features: Transboundary Conservation Efforts Are Needed for the Critically Endangered Balearic Shearwater
Source: PLoS One. 2012 May 10;7(5):e35728. doi: 10.1371/journal.pone.0035728 (PMC3349676; doi:10.1371/journal.pone.0035728)

**Text S1 - Duty cycle transmission**

Argos PTTs had a duty cycle programme (10 hrs on, 48 hrs off) that lead to alternating day-night transmission periods (see the transmission periods for each PTT device in Figure S1.1). After filtering the data, the number of locations per duty cycle ranged between 1 and 9 (Figure S1.2). Indeed, transmission period started around 5 am and 2 pm, while no transmission started between 9 am and 10 am (Figure S1.3). The number of 10-h on periods ranged between 8 and 12 with a range of 1.81 and 5.33 locations per duty cycle lasting between 0 and 10.09 hours (Table 1 & Figure S1.4).

Figure S1.1 Transmission periods of the analysed 4 PTT devices.


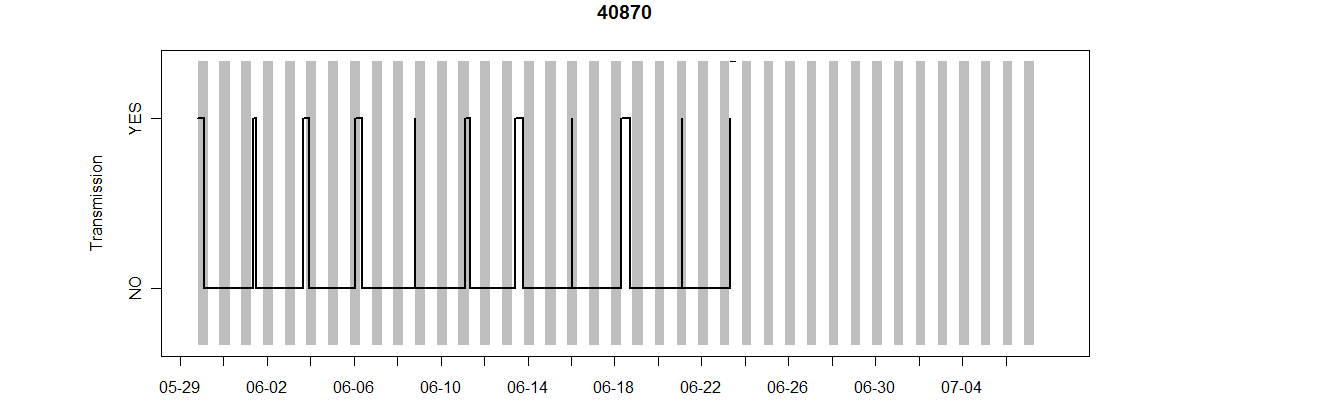

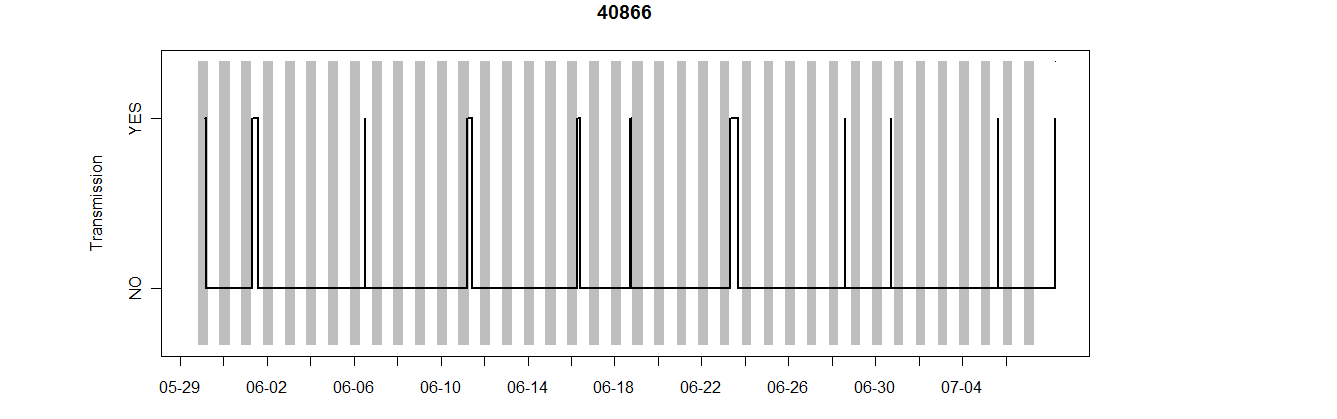

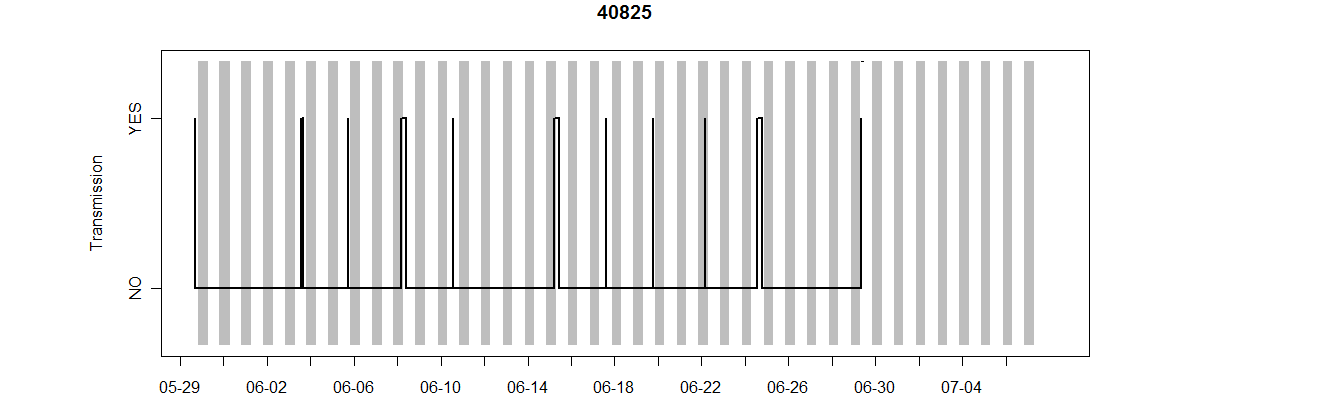

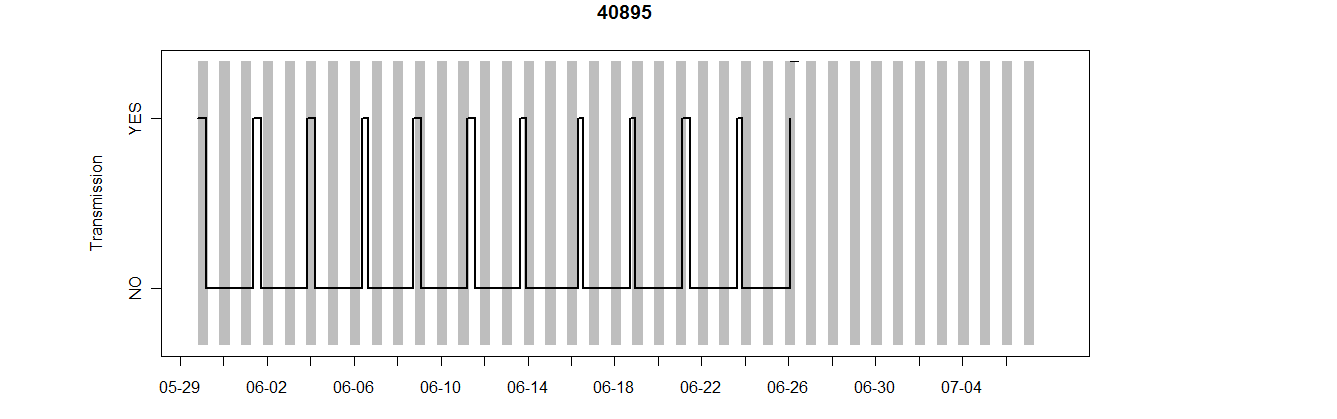


Figure S1.2 Histogram of the number of locations per duty cycle of the analysed 4 PTT devices.


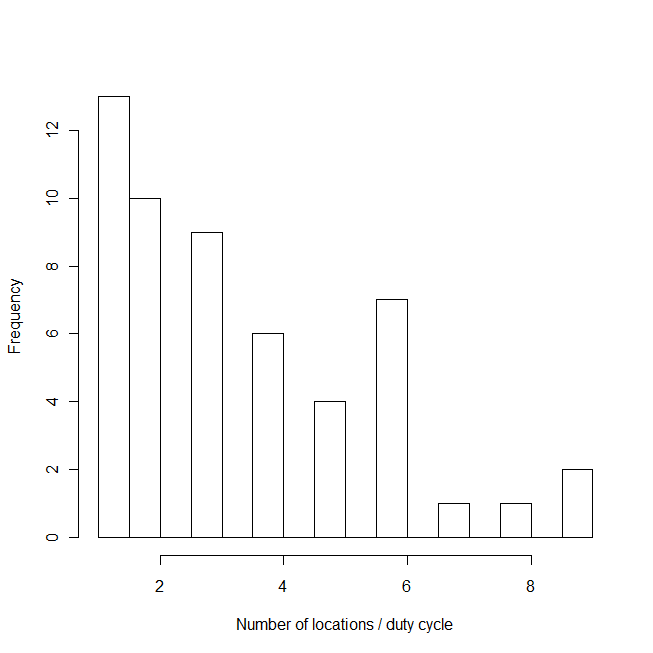


Figure S1.3 Histogram of the hour of the day when the transmission period starts of the analysed 4 PTT devices.


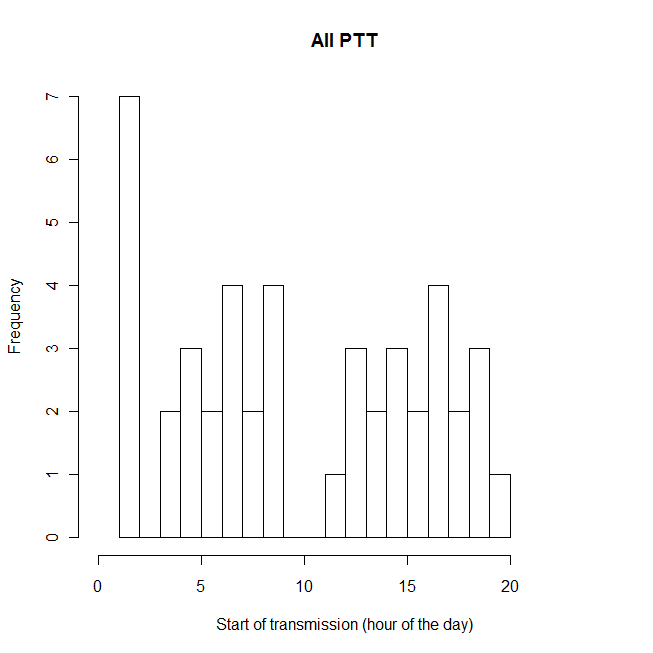


Figure S1.4. Histogram of the transmission duration of the analysed 4 PTT devices.


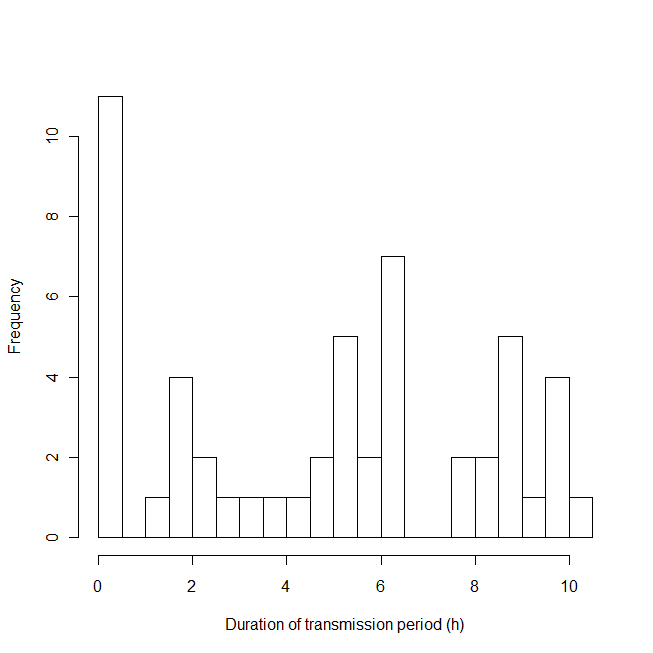

Supplement: Text S1 — Duty cycle transmission. (DOC) [file pone.0035728.s004.doc]
