# Supplementary material for: Protecting Persistent Dynamic Oceanographic Features: Transboundary Conservation Efforts Are Needed for the Critically Endangered Balearic Shearwater
Source: PLoS One. 2012 May 10;7(5):e35728. doi: 10.1371/journal.pone.0035728 (PMC3349676; doi:10.1371/journal.pone.0035728)
Supplement: Text S2 — Choosing a smoothing factor for kernel analysis. (DOC) [file pone.0035728.s005.doc]

**Text S2 - Choosing a smoothing factor for kernel analysis**

We generated density distribution maps using fixed kernel density using the *ad hoc* method of the ‘adehabitat’ package (i.e. bivariate normal kernel; smoothing factor *h* of 0.51) and a cell size of 0.0417° (to match the spatial resolution of the satellite imagery data) in R 2.12.2 [1]. The smoothing factor was chosen based on exploratory analysis comparing the bivariate normal kernel, the least-square cross validation and arbitrarily chosen values (*h* = 1 and *h* = 2). The bivariate normal kernel method showed the best fit to our data of the western Mediterranean basin and was further used for analyses (see figures below).

Figure S2.1. Kernel density estimations the bivariate normal kernel, the least-square cross validation and arbitrarily chosen values (*h* = 1 and *h* = 2).


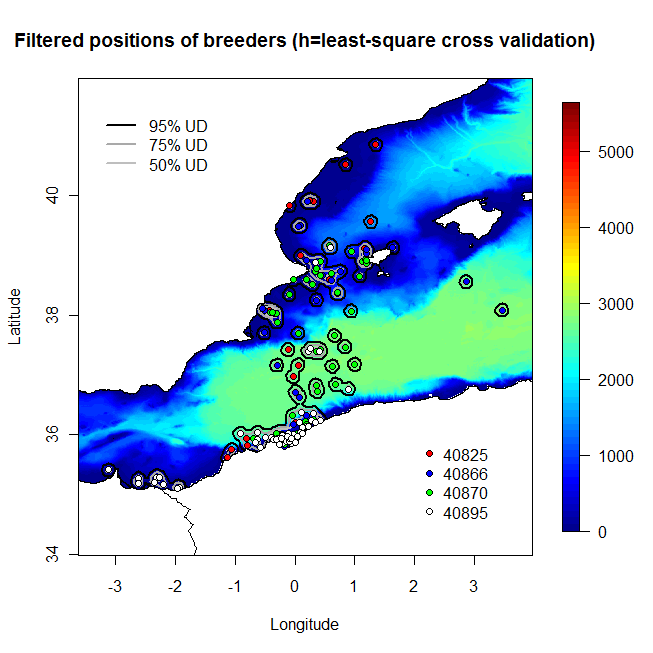

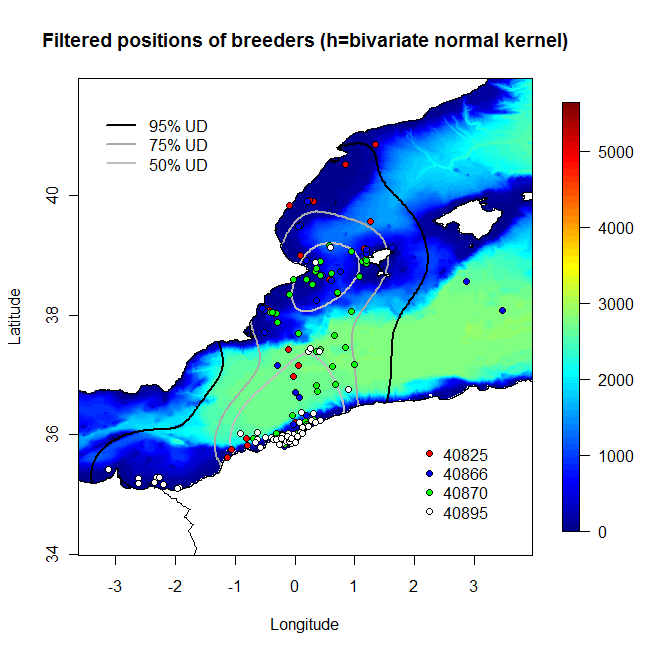

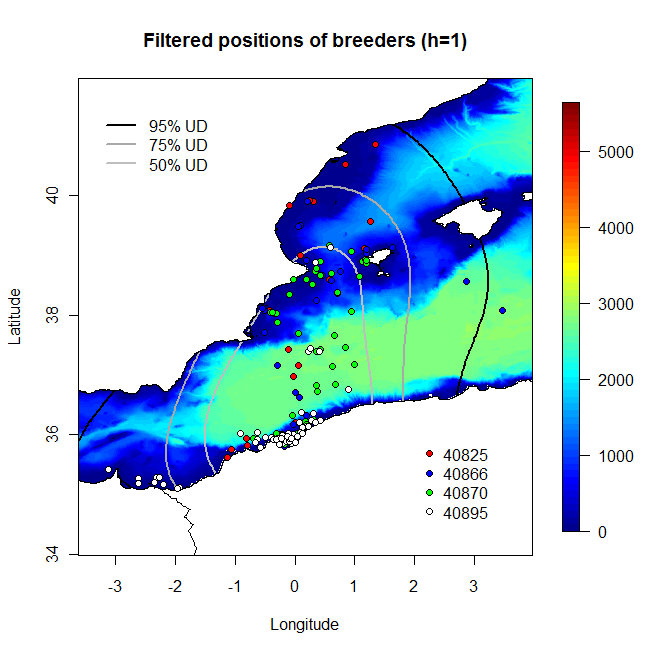

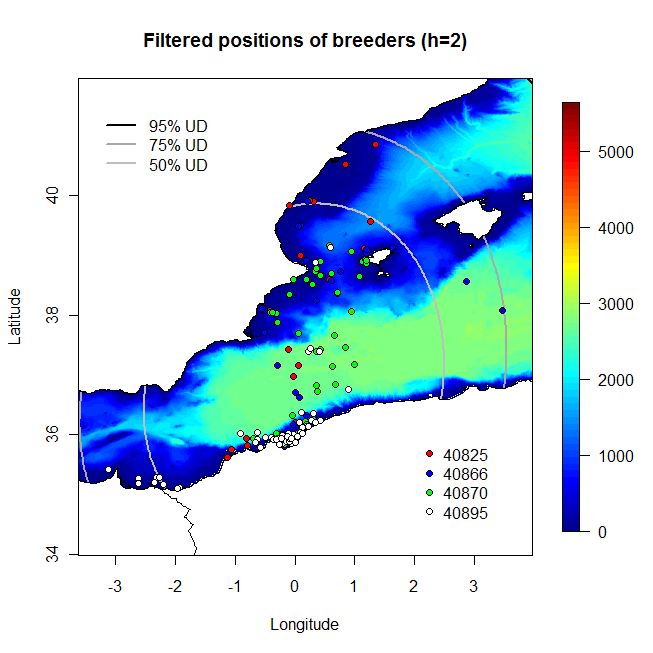


1. Calenge C (2006) The package “adehabitat” for the R software: A tool for the analysis of space and habitat use by animals. Ecological Modelling 197: 516–519.
