# Supplementary material for: Protecting Persistent Dynamic Oceanographic Features: Transboundary Conservation Efforts Are Needed for the Critically Endangered Balearic Shearwater
Source: PLoS One. 2012 May 10;7(5):e35728. doi: 10.1371/journal.pone.0035728 (PMC3349676; doi:10.1371/journal.pone.0035728)

**Text S3 - Effect of duty cycle on kernel estimation**

The duty cycle programme (10 hrs on, 48 hrs off) could influence the kernel density estimates since some locations could be in close proximity during the 10 hr on-cycle and then none for 2 days. When using all locations, kernel estimations could give more emphasis to the areas where the PTT transmitted, rather than properly reflect the proportion of time spent by the birds. In order to test this influence, we compared kernel estimations when using all locations and the central locations (mean latitude and longitude of all locations per duty cycle) of each duty cycle to check whether their size and location would change significantly.

In the figure below, we compared the kernel estimations of the 95% UD, 75% UD and 50% UD estimated using all positions and the mean position of each duty cycle. Both approaches yielded similar position and location of 95% UD, 75% UD and 50% UD (black and grey lines represents kernel density estimations using all locations and mean position per duty cycle, respectively). Thus, we considered that the approach of using all locations was not influenced by the duty cycle programme of the PTT devices.

Figure S3.1. Comparison of kernel estimations of the 95% UD, 75% UD and 50% UD estimated using all positions and the mean position of each duty cycle.


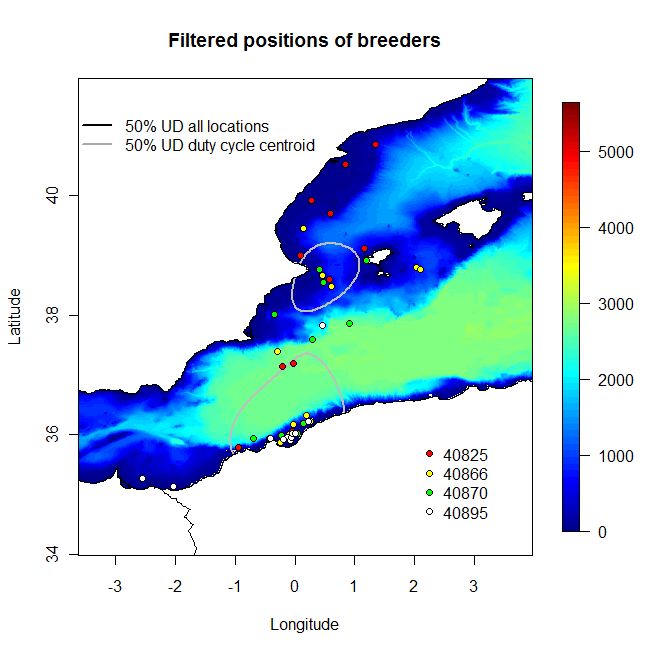

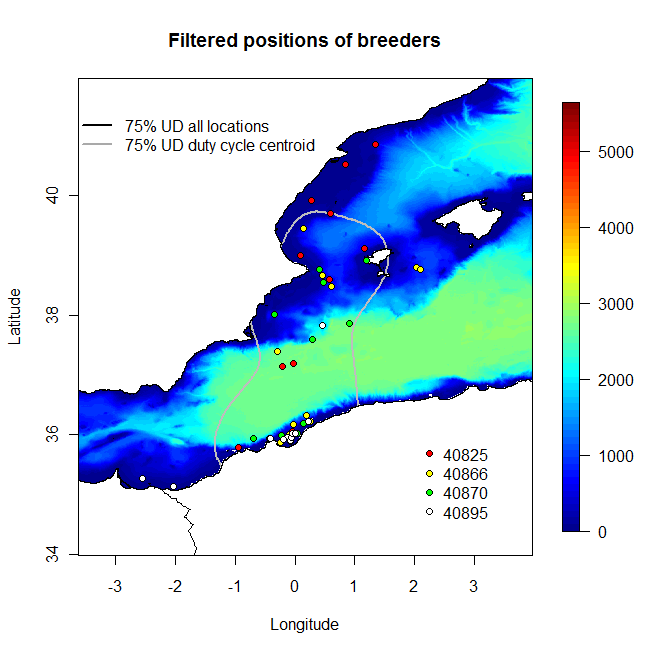

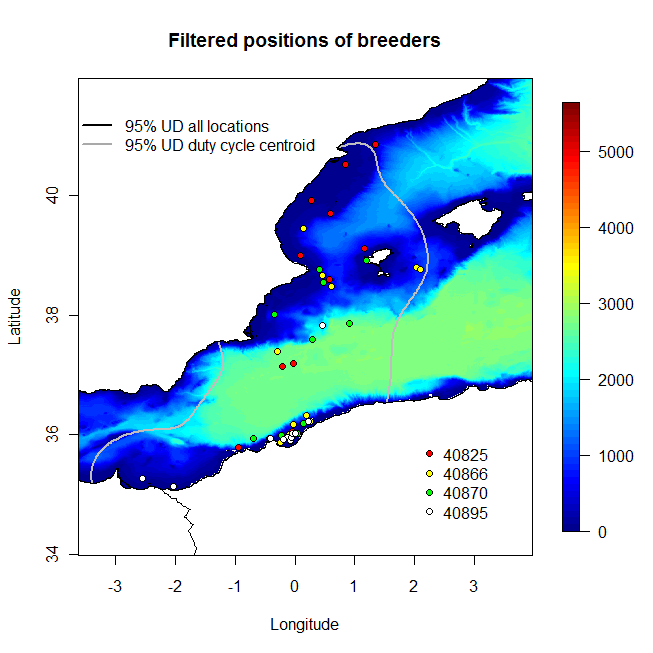


We also studied the influence of the day/night periods on the activity of Balearic shearwaters to exclude the possibility of night-time activity periods heavily influencing the kernel density estimates. Firstly, we have used the threshold of 10 km/h to identify flying and resting (i.e. sitting on the water) periods. We believe that this threshold of travel rates is convincing since GPS-based tracking data on the closely related Cory’s shearwater *Calonectris diomedea* have shown that this threshold can be used to identify those two behaviours (Louzao et al. 2009). Moreover, we defined ‘night’ as the period in which the sun was six degrees or more below the horizon and ‘day’ otherwise using the ‘tripEstimation’ package. Once an activity was assigned to each position, we estimated the percentage of locations flying or resting by bird separately by day and night. We did not find difference on flying and resting during day and night (for flying F_1,8_ = 0.069, P = 0.800; for resting F_1,8_ = 0.069, P = 0.800) and in turn exclude the possibility of night-time activity periods heavily influencing the kernel density estimates (see figure below).

Figure S3.2. Percentage of locations identified as flying or resting separately by day and night for each PTT device.


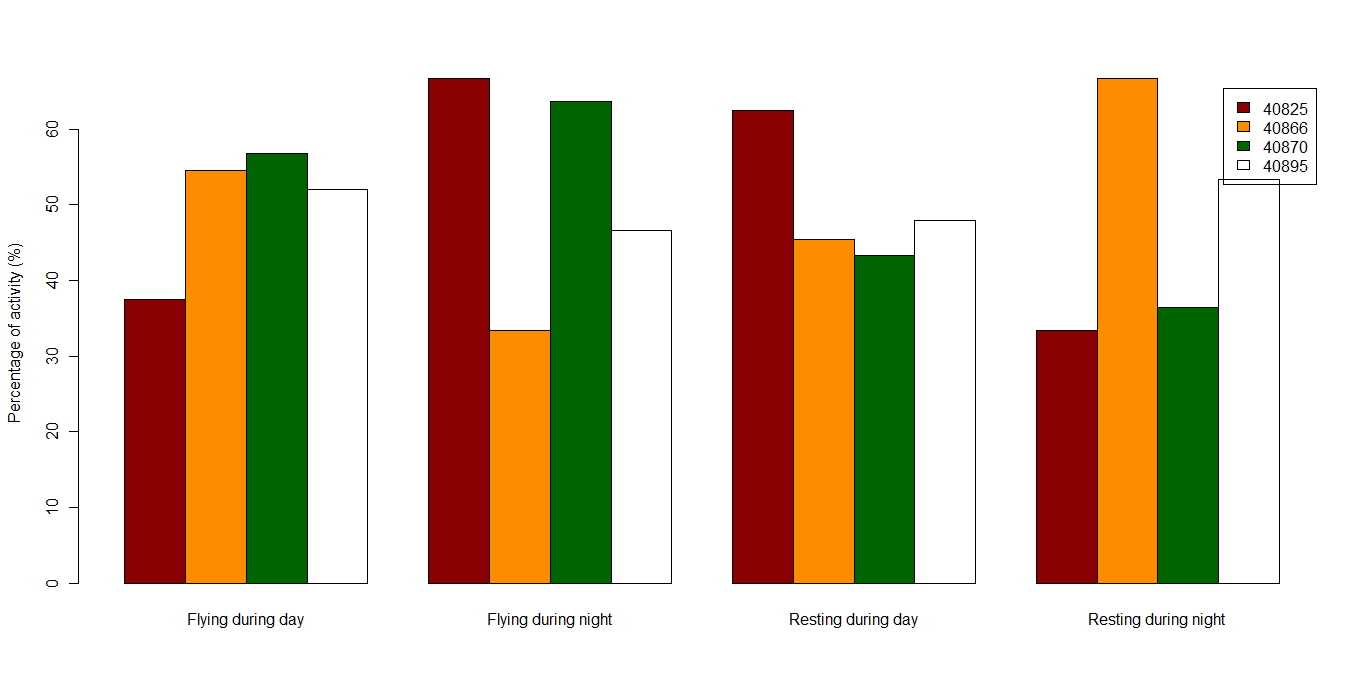

Supplement: Text S3 — Effect of duty cycle on kernel estimation. (DOC) [file pone.0035728.s006.doc]
